# Structured Reporting of Lung Cancer Staging: A Consensus Proposal

**DOI:** 10.3390/diagnostics11091569

**Published:** 2021-08-30

**Authors:** Vincenza Granata, Roberto Grassi, Vittorio Miele, Anna Rita Larici, Nicola Sverzellati, Salvatore Cappabianca, Luca Brunese, Nicola Maggialetti, Andrea Borghesi, Roberta Fusco, Maurizio Balbi, Fabrizio Urraro, Duccio Buccicardi, Chandra Bortolotto, Roberto Prost, Marco Rengo, Elisa Baratella, Massimo De Filippo, Carmelo Barresi, Stefano Palmucci, Marco Busso, Lucio Calandriello, Mario Sansone, Emanuele Neri, Francesca Coppola, Lorenzo Faggioni

**Affiliations:** 1 Division of Radiology, “Istituto Nazionale Tumori IRCCS Fondazione Pascale–IRCCS di Napoli”, 80131 Naples, Italy; v.granata@istitutotumori.na.it; 2 Division of Radiology, “Università degli Studi della Campania Luigi Vanvitelli”, 80128 Naples, Italy; roberto.grassi@unicampania.it (R.G.); salvatore.cappabianca@unicampania.it (S.C.); fabrizio.urraro@unicampania.it (F.U.); 3 Italian Society of Medical and Interventional Radiology (SIRM), SIRM Foundation, Via della Signora 2, 20122 Milan, Italy; vmiele@sirm.org (V.M.); emanueleneri1@gmail.com (E.N.); 4 Division of Radiology, “Azienda Ospedaliera Universitaria Careggi”, 50134 Florence, Italy; 5 Institute of Radiology, Catholic University of the Sacred Heart, Fondazione Policlinico Universitario A. Gemelli, 00168 Rome, Italy; anna.larici@gmail.com; 6 Section of Diagnostic Imaging, Department of Surgery, University of Parma, 43100 Parma, Italy; nicola.sverzellati@unipr.it (N.S.); balbi.m@libero.it (M.B.); 7 Department of Medicine and Health Sciences “V. Tiberio”, University of Molise, Via Francesco De Sanctis 1, 86100 Campobasso, Italy; luca.brunese@unimol.it; 8 Section of Radiodiagnostic, DSMBNOS, “Aldo Moro” University, 70121 Bari, Italy; n.maggialetti@gmail.com; 9 Department of Medical and Surgical Specialties, Radiological Sciences and Public Health, University of Brescia, ASST Spedali Civili of Brescia, Piazzale Spedali Civili, 1, 25123 Brescia, Italy; aborghesi@sirm.org; 10 Medical Oncology Division, Igea SpA, 80013 Naples, Italy; 11 Imaging Department, San Paolo Hospital, 17100 Savona, Italy; buccicardiduccio@hotmail.com; 12 Department of Radiology, I.R.C.C.S. Policlinico San Matteo Foundation, 27100 Pavia, Italy; chandra.bortolotto@gmail.com; 13 Radiology Unit, Azienda Ospedaliera Brotzu, 09134 Cagliari, Italy; rprost@sirm.org; 14 Department of Radiological Sciences, Oncology and Pathology, I.C.O.T. Hospital, “Sapienza”, University of Rome, 00195 Latina, Italy; marco.rengo@gmail.com; 15 Department of Radiology, Cattinara Hospital, University of Trieste, 34128 Trieste, Italy; elisa.baratella@gmail.com; 16 Department of Medicine and Surgery, Unit of Radiologic Science, University of Parma, Maggiore Hospital, 43126 Parma, Italy; massimo.defilippo@unipr.it; 17 Division of Radiology, Azienda Ospedaliera Universitaria Senese, 53100 Siena, Italy; carminev80@alice.it; 18 Department of Medical Surgical Sciences and Advanced Technologies GF Ingrassia, University Hospital Policlinico “G.Rodolico-San Marco”, University of Catania, 95123 Catania, Italy; spalmucci@sirm.org; 19 Radiology Unit, Department of Oncology, San Luigi Gonzaga University Hospital, University of Turin, 10043 Turin, Italy; busso.marco@gmail.com; 20 Department of Diagnostic Imaging, Oncological Radiotherapy, and Hematology–Diagnostic Imaging Area, Fondazione Policlinico Universitario Agostino Gemelli IRCCS, 00168 Rome, Italy; lcalan@hotmail.it; 21 Department of Electrical Engineering and Information Technology (DIETI), University of Naples Federico II, via Claudio 21, 80125 Naples, Italy; msansone@unina.it; 22 Department of Translational Research, University of Pisa, 56126 Pisa, Italy; lfaggioni@sirm.org; 23 Department of Radiology, IRCCS Azienda Ospedaliero-Universitaria di Bologna, Via Albertoni 15, 40138, Bologna, Italy; francesca_coppola@hotmail.com

**Keywords:** radiology report, free text report, structured report, lung cancer, computed tomography

## Abstract

Background: Structured reporting (SR) in radiology is becoming necessary and has recently been recognized by major scientific societies. This study aimed to build CT-based structured reports for lung cancer during the staging phase, in order to improve communication between radiologists, members of the multidisciplinary team and patients. Materials and Methods: A panel of expert radiologists, members of the Italian Society of Medical and Interventional Radiology, was established. A modified Delphi exercise was used to build the structural report and to assess the level of agreement for all the report sections. The Cronbach’s alpha (Cα) correlation coefficient was used to assess internal consistency for each section and to perform a quality analysis according to the average inter-item correlation. Results: The final SR version was built by including 16 items in the “Patient Clinical Data” section, 4 items in the “Clinical Evaluation” section, 8 items in the “Exam Technique” section, 22 items in the “Report” section, and 5 items in the “Conclusion” section. Overall, 55 items were included in the final version of the SR. The overall mean of the scores of the experts and the sum of scores for the structured report were 4.5 (range 1–5) and 631 (mean value 67.54, STD 7.53), respectively, in the first round. The items of the structured report with higher accordance in the first round were primary lesion features, lymph nodes, metastasis and conclusions. The overall mean of the scores of the experts and the sum of scores for staging in the structured report were 4.7 (range 4–5) and 807 (mean value 70.11, STD 4.81), respectively, in the second round. The Cronbach’s alpha (Cα) correlation coefficient was 0.89 in the first round and 0.92 in the second round for staging in the structured report. Conclusions: The wide implementation of SR is critical for providing referring physicians and patients with the best quality of service, and for providing researchers with the best quality of data in the context of the big data exploitation of the available clinical data. Implementation is complex, requiring mature technology to successfully address pending user-friendliness, organizational and interoperability challenges.

## 1. Introduction

The American Recovery and Reinvestment Act and the Health Information Technology for Economic and Clinical Health Act have indicated that structuring data in health records will lead to an important improvement in patient outcomes [1,2]. Since the radiology report is part of the health record, the current format of free-text reporting (FTR) should be organized and shifted toward structured reporting (SR). The issue of whether all radiological examinations should contain a structured report, and if so, what the actual report structure should be, remains open [1,2,3]. According to the European Society of Radiology’s (ESR) paper on SR in radiology [1], the three main reasons for moving from FTR to SR are quality, data quantification and accessibility. A critical quality improvement dimension resulting from the use of SR is standardization. The use of templates in SR provides a checklist as to whether all relevant items for a particular examination have been addressed. Thanks to this “structure”, the radiology report will also allow the association of radiological data and other key clinical features, leading to a precise diagnosis and personalized medicine. With regards to accessibility, it is known that radiology reports are a rich source of data for research. This allows automated data mining, which may help to validate the relevance of imaging biomarkers by highlighting the clinical contexts in which they are most appropriate, and to devise potential new application domains. For this reason, radiology reports should be structured via their content, based on standard terminology, and should be accessible via standard access mechanisms and protocols. 

Weiss et al. have described three levels of SR [4]:

1. The first level is a structured format with paragraphs and subheadings. Currently, almost all radiology reports display this structure, with sections for clinical information, examination protocol and radiological findings, and a conclusion to highlight the most important findings.
2. The second level refers to consistent organization. For example, rectal cancer magnetic resonance imaging (MRI) describes all relevant features, such as tumor (T) stage, node (N) stage, anal sphincter complex involvement, tumor deposits in the mesorectal space, extramural vascular invasion, etc.
3. The third level directly addresses the consistent use of dedicated terminology, namely, standard language.

Several proposals have been made by major International Societies of Radiology to support the use of SR [5,6,7,8,9,10]. The Italian Society of Medical and Interventional Radiology (SIRM) has created an Italian warehouse of SR templates, which can be freely accessed by all SIRM members, for the purpose of being routinely used in a clinical setting [11]. 

Despite these promising developments, SR has not yet been established in clinical routine. A survey of Italian radiologists found that the majority of those surveyed had heard of SR, but only a minority of them regularly used it in their clinical work [10]. Reasons for this include the current lack of usable templates and the minimal availability of software solutions for SR [10].

Lung cancer is the leading cause of cancer morbidity and mortality in men, whereas in women, it ranks third for incidence after breast and colorectal cancer, and second for mortality after breast cancer [12]. The incidence and mortality rates are roughly twice as high in men than in women, although the male-to-female ratio varies widely across regions. Lung cancer incidence and mortality rates are 3 to 4 times higher in transitioned countries than in transitioning countries; this pattern may well change as the tobacco epidemic evolves, given that 80% of smokers aged 15 years or older resided in low-income and middle-income countries in 2016 [12,13]. In the absence of symptoms to identify early lung cancer, screening high-risk individuals has the potential of shifting the diagnosis to earlier stages [14,15,16,17,18,19,20]. After more than 30 years of research, a large randomized controlled trial established that low-dose computed tomography (CT) improved mortality in patients at high risk for lung cancer. Subsequently, the majority of professional societies emphasize the importance of lung cancer screening. Although lung cancer screening is not unanimously recommended, the value of identifying early-stage lung cancer cannot be overstated. The majority of new cases of lung cancer present in advanced stages (III–IV), when a cure is unlikely or unattainable [21]. 

In this context, a disease-specific SR could be an effective tool for conveying all diagnostic imaging information needed for a correct lung cancer diagnosis and staging, while including clinical information required for personalized patient management.

The aim of the present study was to propose an SR template that can guide radiologists in the systematic reporting of CT examinations for lung cancer staging, in order to improve communication between radiologists and clinicians, particularly in non-referral centers.

## 2. Materials and Methods 

### 2.1. Panel Expert

As a result of critical discussion between expert radiologists, a multi-round consensus-building Delphi exercise was carried out to develop a comprehensive and focused SR template for CT staging of patients with lung cancer. 

A SIRM radiologist expert in thoracic imaging created the first draft of the SR template for lung cancer staging CT examinations. 

A working team of 13 experts from the Italian College of Thoracic Radiologists and of Diagnostic Imaging in Oncology Radiologists from SIRM was established to iteratively revise the initial draft, with the aim of reaching a final consensus on SR.

### 2.2. Selection of the Delphi Domains and Items 

All the experts reviewed the literature data on the main scientific databases (including Pubmed, Scopus and Google Scholar) from December 2000 to December 2020, in order to assess papers on lung cancer CT and radiological SR. The full text of the studies selected was reviewed by all members of the expert panel, and each of them developed and shared the list of Delphi items via email and/or teleconference. 

The SR was divided into five sections: (1) Patient Clinical Data, (2) Clinical Evaluation, (3) Exam Technique, (4) Report and (5) Conclusion. A dedicated section for key images was added as part of the report.

1. The “Patient Clinical Data” section included patient clinical data and previous or family history of malignancies, including previous lung cancer, risk factors or predisposing pathologies. In this section, the item of “Allergies” to drugs and contrast medium was included.
2. The “Clinical Evaluation” section included previous examination results, a genetic panel and clinical symptoms.
3. The “Exam Technique” section included data regarding the CT equipment used (including the number of detector rows and whether single or dual energy scans were performed) and information concerning reconstruction algorithm(s) and slice thickness. Data on the contrast protocol were also collected (including information regarding post-contrast acquisitions), as well as data concerning the contrast medium (such as contrast active principle, commercial name, volume, flow rate, iodine concentration, and ongoing adverse events).
4. The “Report” section included data regarding lung cancer location, morphology, margin sharpness, texture (e.g., solid, ground glass), contrast enhancement pattern, size, local invasion, tumor stage, node stage and metastatic stage, according to the Italian Association of Medical Oncology (AIOM) guidelines [22]. In this section, a dedicated subsection for other types of primary lung cancers was included.
5. The “Conclusion” section included diagnosis, TNM stage according to the 8th Edition of AJCC-UICC 2017 [23], annotations and comments.

Two Delphi rounds were carried out [24]. During the first round, each panelist independently contributed to refining the SR draft by means of online meetings or email exchanges. The level of panelist agreement for each SR model was tested in the second Delphi round, using a Google Form questionnaire shared by email. Each expert made individual comments for each specific template part (i.e., patient clinical data, clinical evaluation, exam technique, report and conclusion, images) using a five-point Likert scale (1 = strongly disagree, 2 = slightly disagree, 3 = slightly agree; 4 = modestly agree, 5 = strongly agree). 

After the second Delphi round, the final version of the SR was generated on the dedicated Radiological Society of North America (RSNA) website (radreport.org), using a T-Rex template format in line with the IHE (Integrating the Healthcare Enterprise) and MRRT (Management of Radiology Report Templates) profiles, accessible as open-source software, with the technical support of Exprivia™. These determined both the format of the radiology report templates (using version 5 of the Hypertext Markup Language (HTML5)) and the transporting mechanism used to request, get back and stock these schedules [25]. The radiology report was structured using a series of “codified queries” integrated into the T-Rex editor’s preselected sections [25]. 

### 2.3. Statistical Analysis

All ratings of the panelists for each section were analyzed using descriptive statistics measuring the mean score, the standard deviation value (STD) and the sum of scores. A mean score of 3 was considered good and a score ≥ 4 excellent. 

To measure the internal consistency of the panelist ratings for each section of the report, a quality analysis based on the average inter-item correlation was carried out using the Cronbach’s alpha (Cα) correlation coefficient [26,27]. The Cα test provides a measure of the internal consistency of a test or scale; it is expressed as a number between 0 and 1. Internal consistency describes the extent to which all the items in a test measure the same concept. The Cα correlation coefficient was determined after each round.

The closer to 1.0 the Cα coefficient, the greater the internal consistency of the items in the scale. An alpha coefficient (α) > 0.9 was considered excellent, α > 0.8 good, α > 0.7 acceptable, α > 0.6 questionable, α > 0.5 poor, and α < 0.5 unacceptable. However, in the iterations, an α of 0.8 was considered to be a reasonable goal for internal reliability.

The data analysis was carried out using Statistic Toolbox of Matlab (The MathWorks, Inc., Natick, MA, USA).

## 3. Results

### 3.1. Structured Report 

The final SR (Appendix A) version was built by including 16 items in the “Patient Clinical Data” section, 4 items in the “Clinical Evaluation” section, 8 items in the “Exam Technique” section, 22 items in the “Report” section, and 5 items in the “Conclusion” section. Overall, 55 items were included in the final version of the SR. In Appendix B, the first draft of the SR is illustrated.

The results obtained during the first Delphi round are reported in Table 1, and those obtained after the second Delphi round in Table 2. 

In the final version of the SR, the following parameters were included:

1. In the “Exam technique” section, the equipment used, the number of detector rows and CT modality (i.e., single or dual energy), the reconstruction algorithm(s) used and contrast protocol;
2. In the “Report” section, the sites and the features of extrathoracic metastases were defined, identifying the target lesions in accordance with the Response Evaluation Criteria in Solid tumors (RECIST) 1.1 [28].

### 3.2. Consensus Agreement

Table 1 and Table 2 show the single scores and the sums of scores of the panelists for staging with the SR in the first and second rounds, respectively. 

In both the first and the second rounds, as reported in Table 1 and Table 2, all sections received more than a good rating. 

The overall mean score of the experts (13 experts) and the sum of scores for staging with the SR were 4.5 (range 1–5) and 631 (mean value 67.54, STD 7.53) (Table 1), respectively, in the first round. The items of the SR with higher accordance in the first round were primary lesion features, lymph nodes, metastases and conclusions (Table 1).

The overall mean score of the experts (nine experts) and the sum of scores for staging with the SR were 4.7 (range 4–5) and 807 (mean value 70.11, STD 4.81) (Table 2), respectively, in the second round. 

The overall mean score of the experts in the second round was higher than the overall mean score of the experts in the first round, with a lower standard deviation value demonstrating the higher agreement reached among the experts in the SR in this round. The items of the SR in the second round that had higher “each reader” accordance were exam data and pulmonary involvement in multiple sites (Table 2).

The Cronbach’s alpha (Cα) correlation coefficient was 0.89 in the first round and 0.92 in the second round for staging with the SR. 

## 4. Discussion 

In the present study, the panel of experts demonstrated a high degree of agreement in defining the different items of the SR. After the second Delphi round, the panelists’ mean score and the sum of scores related to the SR models were 4.7 (range 4–5) and 807 (mean value 70.11, STD 4.81), respectively. All sections received more than a good rating in the second Delphi round; however, the weakest sections were “Patient Clinical Data” and “Clinical Evaluation”. Moreover, the Cα correlation coefficient reached 0.92 in the second round. 

The present SR is based on a multi-round consensus-building Delphi exercise performed to develop a comprehensive focus on the SR template for CT-based lung cancer staging, as a result of critical discussion between expert radiologists in thoracic and oncological imaging. This SR was based on a standardized terminology and structure, which are aspects required for adherence to diagnostic-therapeutic recommendations and for enrolment in clinical trials, thus reducing the ambiguity that may arise from non-conventional language, and enabling better communication between radiologists and clinicians [29,30,31,32,33]. Therefore, according to Weiss et al. [4], the present report is a third-level SR.

Several sections are included in the present template: “Patient Clinical Data”, “Clinical Evaluation”, “Exam Technique”, “Report” and “Conclusion”. Some points should be evaluated for each of these sections. 

Regarding “Patient Clinical Data”, this section included data regarding personal or family history of cancer, and exposure to different risk factors and any genetic mutations. Regarding predisposing diseases, the possibility of collecting data on Chronic Obstructive Pulmonary Disease (COPD) allows one to plan treatment tactics. COPD is generally defined as a chronic minimally reversible airflow obstruction based on spirometry (post-bronchodilator forced expiratory volume in 1 second (FEV1)/forced vital capacity (FVC) less than 70%).

COPD and lung cancer share common features, including their high mortality and common risk factors (such as smoking), some genetic background, environmental exposures, and underlying common inflammatory processes. A ratio of FEV1 to FVC less than 0.7 is generally used to define airflow obstruction; however, other indices (such as FEV1/FVC under the lower limit of normal criteria, and a predicted reduction of FEV1%) have also been considered indicative of airway obstruction. In addition to these three main factors, the timing of COPD diagnosis, the degree of airflow obstruction, and the severity of emphysema have also been reported to exert a remarkable effect on the significance of the impact of COPD and/or emphysema on lung cancer risk. Although, at present, no solid evidence is available to clearly distinguish the roles of airflow obstruction and emphysema in lung cancer development, it is certain that the highest lung cancer risk occurs when airflow obstruction and emphysema coexist [12,13,14].

Such a painstaking process of data collection was subject to some disagreement among the panelists due to the opinion that this process could slow down the normal workflow and was not considered to be easy to use. However, it is necessary to point out that all SR sections are independent from each other, so that the Patient Clinical Data and Clinical Evaluation sections are optional and may be filled in or not upon user choice, although they were conceived with the aim of creating databases. In fact, the possibility of collecting all these data could allow the creation of a large database, not only for epidemiological studies, but also in the highest conception of radiology, to lay the foundations for radiomics studies [34,35,36,37]. Radiology reports should be rich in data that could potentially be pooled, analyzed and correlated with patient outcomes, thereby assisting future clinical and imaging guidelines. However, the use of non-standardized terminology limits the capacity for data collection across multiple institutions. In addition, the lack of consistent data extractable from SR could hinder the development of computerized applications to assist in reporting. Natural language processing applications can help extract the data from the reports with variable terminology, allowing the compilation of standardized data, which could then be used to develop multi-institutional data registries, as well as in clinical and research analyses. Moreover, the possibility of combining genomic data and radiological features allows for developing models of radiogenomics—models that today represent the highest level of advanced-precision medicine processes [38,39,40,41]. The fact that the present SR can be included in the picture archiving and communication system (PACS) is an added value; therefore, it is only necessary to enter these data once upon first entry into the radiology department.

With regard to the “Exam Technique” section, sharing the examination technique not only within one’s own department, but also with the radiology departments of other centers, fulfills a dual purpose. On the one hand, it enables the standardization of CT protocols; on the other hand, it allows carrying out diagnostic accuracy studies among different centers in order to optimize CT protocols. For example, during follow-up, differences in CT acquisition parameters and segmentation algorithms are important factors that can lead to variability in volumetric measurements. Therefore, slice thickness and other protocol-related factors (such as the reconstruction kernel and field of view) should be kept constant for reliable measurements to be carried out. Although some software packages allow the customization of options (which changes density thresholds for segmentation), standardized parameters should exist between practices in order to keep these parameters homogenous and comparable. In the CT protocol optimization step, enhanced communication among different centers could theoretically lead to quality improvement by means of enhanced patient safety (e.g., by radiation dose reduction), contrast optimization, and image quality. With improved communication comes the sharing of knowledge and experience, along with the potential of reducing medical errors and improving clinical outcomes [42].

Some authors have reported that the use of a checklist could improve diagnostic accuracy [43,44,45]. In 2014, based on the results of several screening trials, the American College of Radiology (ACR) released version 1.0 of the Lung CT Screening Reporting and Data System (Lung-RADS) [46]. This is a standardized method of reporting with recommendations for the management of pulmonary nodules detected on CT for lung cancer screening. When utilized, it can reduce the false positive rate in lung cancer screening, without increasing the rates of false negatives [47,48]. Lung-RADS is now deeply embedded as a quality metric on which regulation and reimbursement is determined by the Centers for Medicare and Medicaid [49,50]. During the first 5 years of nationwide lung cancer screening, there was a significant accumulation of data and experience, with many opportunities for continued learning [49,50]. 

The present “Report” section was designed to report all the structural characteristics of the lesions, such as margins and density, as well as relationships with locoregional structures (e.g., the pleura), which allow correct staging, but could also impact the choice of a more suitable therapeutic treatment based on the individual patient. The advantages of SR over FTR include its standardized terminology and structure, aspects required for adherence to diagnostic-therapeutic recommendations and for enrolment in clinical trials. SR reduces the ambiguity that may arise from non-conventional language. However, it should be noted that SR templates usually include a free text box for reporting any additional data that cannot be embedded in the default template fields. 

The wide implementation of SR is critical for providing referring physicians and patients with the best quality of service, and for providing researchers with best quality data in the context of the big data exploitation of available clinical information [51,52,53,54]. Implementation is complex, requiring mature technology to successfully address pending user-friendliness, organizational and interoperability challenge (with particular regard to the adequate storage of data, and easy and adequate connections with PACS and post-processing software). Consequently, the introduction of SR should be seen as a comprehensive effort, affecting all domains of radiology [55,56,57,58].

Despite the promising results obtained, this study has some limitations. First, the panelists were all radiologists; therefore, a multidisciplinary approach is lacking. A multidisciplinary validation of SR would have been more appropriate. Second, the panelists were of the same nationality; contributions from experts from multiple countries would allow for broader sharing, and would increase the consistency of the SR. Finally, this study was not aimed at assessing the impact of SR on the management of patients with lung cancer. This issue will be discussed in forthcoming studies. 

## 5. Conclusions

The wide implementation of SR is a critical point for providing referring physicians and patients with the best quality of service, and for providing researchers with the best quality of data in the context of the big data exploitation of the available clinical information. Implementation is complex, requiring mature technology to successfully address pending user-friendliness, organizational and interoperability challenges (specifically, the adequate storage of data, and the easy and adequate connection with PACS and post-processing software). Consequently, the introduction of SR should be seen as a comprehensive effort, affecting all domains of radiology.

The authors have no conflict of interest to disclose. The authors confirm that the article is not under consideration for publication elsewhere. Each author participated sufficiently to take public responsibility for the content of the manuscript.

## Figures and Tables

**Table 1:** Single scores and the sums of scores of panelists for staging in the SR (round I).

P #	A1. Anthropometric Data	A2. Personal Assessments	A3. Allergies	B1. Clinical Data	C1. Exam Data	C2. Contrast Medium	C3. Adverse Events	D1. Primary Lesion Features	D2. Loco-Regional Extension	D3. Lymph Nodes	D4. Metastasis	D5. Multiple-Site Pulmonary Involvement	D6. Other Findings	E1. Conclusions	Meaningful Key Images	Sum of Scores
1	5	5	5	5	5	5	5	5	5	5	5	5	5	5	5	75
2	1	2	5	4	3	3	5	5	5	5	5	3	5	4	5	60
3	4	4	3	3	3	3	3	3	3	3	4	3	3	4	4	50
4	5	3	4	5	3	5	4	4	4	5	5	4	5	4	5	65
5	5	5	5	5	5	5	5	5	5	5	5	5	5	5	5	75
6	5	5	5	5	5	5	5	5	5	5	5	5	5	5	5	75
7	5	5	5	5	3	5	5	5	5	5	5	5	5	5	5	73
8	2	5	2	4	3	3	3	5	5	5	5	5	3	5	5	60
9	4	5	5	5	5	5	5	5	5	5	5	5	5	5	4	73
10	4	4	5	4	3	5	5	5	3	4	5	5	5	4	5	66
11	4	4	4	5	5	4	5	5	5	5	5	4	5	5	5	70
12	5	5	4	5	5	5	5	4	5	4	4	5	5	5	5	71
13	3	3	4	4	4	4	4	5	5	5	5	5	4	5	5	65
Mean	4.00	4.23	4.31	4.54	4.00	4.38	4.54	4.69	4.62	4.69	4.85	4.54	4.62	4.69	4.85	67.54
Std	1.29	1.01	0.95	0.66	1.00	0.87	0.78	0.63	0.77	0.63	0.38	0.78	0.77	0.48	0.38	7.53

Note STD: standard deviation value; CM: contrast medium.

**Table 2:** Single scores and sums of scores of panelists for staging in the SR (round II).

Panelist #	A1. Anthropometric Data	A2. Personal Assessments	A3. Allergies	B1. Clinical Data	C1. Exam Data	C2. Contrast Medium	C3. Adverse Events	D1. Primary Lesion Features	D2. Loco-Regional Extension	D3. Lymph Nodes	D4. Metastasis	D5. Multiple-Site Pulmonary Involvement	D6. Other Findings	E1. Conclusions	Meaningful Key Images	Sum of Scores
1	4	5	5	4	5	5	5	5	5	5	5	5	5	5	5	73
2	5	5	4	5	4	4	4	4	4	4	4	4	4	4	5	64
3	5	4	4	4	5	4	4	4	4	4	4	5	4	4	5	64
4	5	4	4	4	5	4	4	4	4	4	4	5	4	4	5	64
5	4	4	5	5	5	5	5	5	5	5	5	5	5	5	5	73
6	5	5	5	5	5	5	5	5	5	5	5	5	5	5	5	75
7	4	4	5	5	5	5	5	5	5	5	5	5	5	5	5	73
8	4	4	5	5	5	5	5	5	4	4	5	5	5	4	5	70
9	5	5	5	5	5	5	5	5	5	5	5	5	5	5	5	75
Mean	4.56	4.44	4.67	4.67	4.89	4.67	4.67	4.67	4.56	4.56	4.67	4.89	4.67	4.56	5.00	70.11
Std	0.53	0.53	0.50	0.50	0.33	0.50	0.50	0.50	0.53	0.53	0.50	0.33	0.50	0.53	0.00	4.81

Note STD: standard deviation value; CM: contrast medium.

## Data Availability

All data are reported in this manuscript.

## Appendix A

**Table A1:** Patient’s clinical data (imported from RIS).

FIELD	DETAIL	ADMITTED VALUES
ANTHROPOMETRIC DATA		
**Weight**		[Numeric] (kg)
**Height**		[Numeric] (cm)
**BMI**		[Numeric] (automatically calculated)
**BSA**		[Numeric] (automatically calculated)
**Age**		[Numeric] (years)
**Age range**		0–49 50–69 ≥70

**Table A2:** Patient’s history.

**Family history of lung cancer** **(visible only if “Yes” and repeatable)**		**Yes/No**
	Degree of kinship	Mother (1st degree) Father (1st degree)
	Notes	[Free text]
**Family history of cancer** **(visible only if “Yes” and repeatable)**		Yes/No
	Degree of kinship	Mother (1st degree) Father (1st degree)
	Notes	[Free text]
**Predisposing diseases** **(visible only if “Yes” and repeatable)**		Yes/No
	Type	Emphysema COPD (ratio of FEV1 and FVC; timing of COPD diagnosis, degree of airflow obstruction and severity of emphysema) Idiopathic pulmonary fibrosis Familial fibrosis Fibrosis in connectivitis Previous cancer (incl. lung cancer, >5 years) Previous chest radiation therapy
	Notes	[Free text]
**Previous lung cancer** **(visible only if “Yes” and repeatable)**		Yes/No
	Histopathological classification (WHO 2015)	Adenocarcinoma Squamous cell carcinoma Large cell carcinoma Neuroendocrine tumors: - Small-cell lung carcinoma; - Large-cell neuroendocrine carcinoma; - Typical carcinoid; - Atypical carcinoid Adeno-squamous carcinoma Sarcomatoid carcinoma
	pT, N, M, Stage yT, N, M, Stage AJCC-UICC Classification (8th Edition, 2017)	
	Notes	[Free text]
**Risk factors**		Yes/No
	Type	Age (≥50 years old) Smoke Secondhand smoke Environmental exposure to the following: - Asbestos; - Radon; - Uranium; - Silica; - Cadmium; - Arsenic; - Beryllium; - Chromium; - Nickel; - Polycyclic aromatic hydrocarbons; - Ethylene oxide; - Benzene; - Coal smoke; - Chloromethylether; - Vinyl chloride; - Fine powders (particulate matter: PM10, PM2.5)
	SMOKING DETAILS	
	Smoker (visible only if “Smoke” selected)	Current smoker Former smoker
	Cigarette smoke	Yes/No
	Number of daily cigarettes (if current smoker)	Light (<15) Heavy (≥15)
	Smoking years	[Numeric]
	Years from cessation (if former smoker)	≤15 >15
	Cigarettes per year (pack-year) (if former or current smoker)	[Numeric] (automatically calculated) (Number of daily cigarettes x smoking years/20)
	Risk	Low risk (<50 years old and/or <20 packs per year of smoking) Moderate risk (≥50 years old and ≥20 packs per year of active or passive smoking, without other risk factors) High risk **group 1** (≥50 years old and ≥20 packs per year of smoking history, in presence of another risk factor (except secondhand smoking)) **group 2** (55–77 years old and ≥30 packs per year of active smoking and smoking cessation <15 years) **Source: NCCN guidelines v. 1.2020**
	Vaping	Yes/No
	Number of daily electronic cigarettes refills (if vaping = Yes0	[Numeric]
	Number of years (if vaping = Yes)	[Numeric]
	Notes	[Free text]
**ALLERGY/PREVIOUS OR ONGOING ADVERSE REACTIONS**		
**Reported allergies** **(visible only if “Yes”)**		Yes/No
	Type	Drug-related (n of drugs) Contrast medium-related (n of contrast media) Drug-unrelated
**(repeatable n times, for all n drugs and/or contrast media specified in “Type”)**	Active principle/molecule (if drug- or contrast medium-related allergy)	[free text]
	Commercial name (if drug- or contrast medium-related allergy0	[free text]
	Notes	[free text]
**(visible if “Drug unrelated” type)**	Urticaria	Yes/No
	Triggering agent	[free text]
	Notes	[free text]
**Ongoing allergy**		Yes/No
**(visible only if “Yes”)**		Ongoing urticaria Other [free text]
**PREVIOUS adverse reactions** **(visible only if “Yes” and repeatable)**	Yes/No	
	Date	month/year (mm/yyyy)
	Type	Contrast medium-related/unrelated
	Degree	Mild Moderate Severe
	Time of onset	Early Late
	Notes	[free text]
**Antiallergic premedication**		Yes/No
	Treatment	Steroid Antihistamine
	Complete	Yes/No
	Notes	[free text]
**Nephroprotective protocol**		Yes/No
	Complete	Yes/No
	Serum creatinine	[Numeric] (mg/dL)
	GFR (glomerular filtration rate)	[Numeric] (mL/min) https://www.merckmanuals.com/medical-calculators/GFR_CKD_EPI-it.htm accessed on 20 April 2021 (sex, race, age, serum creatinine level)
	Notes	[free text]

**Table A3:** Clinical evaluation.

FIELD	DETAIL	ADMITTED VALUES
CLINICAL INFORMATION		
**Prior examinations** **(visible only if “Yes”)**		Yes/No
	Note	[free text]
**Biological work-up**		Yes/No
	Detail	EGFR mutation KRAS mutation ALK rearrangement (fusion) ROS1 rearrangement (fusion) RET rearrangement (fusion) BRAF V600 mutation PDL-1 expression Immunohistochemistry: TTF-1 CK7 (cytokeratin) Napsin p63, p40 CK5/6 Desmocollin-3 Chromogranin Synaptophysin
**Symptoms**	Type	SPECIFIC SYMPTOMS (for primary tumor) Cough Shortness of breath Chest pain Hemoptysis NONSPECIFIC SYSTEMIC SYMPTOMS Anorexia Weight loss Fatigue Fever SYMPTOMS RELATED TO METASTATIC SITES [Free text]
	Notes	[Free text]
**Clinical indication**	Primary staging	Yes/No

**Table A4:** Imaging protocol.

FIELD	DETAIL	ADMITTED VALUES
IMAGING DATA		
**Date of examination**		[Date]
**Scanner brand and model**		[Free text]
**Scanning technique**	Number of detector rows	[Numeric]
	Precontrast scan	Yes/No
**(visible only if “Precontrast scan: Yes”)**		Dual energy (Yes/No)
		Slice thickness (mm) [numeric]
		Slice thickness (mm) [numeric]
**Imaging protocol**		
**(details repeated for each post-contrast scan)**	Pulmonary CT angiography Triple-phase liver acquisition Arterial phase (chest) Venous phase (chest)	Yes/No Yes/No Yes/No Yes/No
**Technical parameters**		
		Dual energy (Yes/No)
		Slice thickness (mm) [numeric]
		Slice thickness (mm) [numeric]
	Notes	[Free text]
**Class of radiation exposure**		[Numeric]
**CONTRAST MEDIUM**		
**Use of contrast medium** **(visible only if “Yes”)**		Yes/No
	Active principle	[Name of active principle of contrast medium]
	Commercial name	[Free text]
	Volume	[Numeric] (mL)
	Flow rate	[Numeric] (mL/s)
	Concentration	[Numeric] (mg I/mL)
	Notes	[Free text]

**Table A5:** ADVERSE EVENTS.

FIELD	DETAIL	ADMITTED VALUES
**ONGOING adverse events** **(visible only if ”Yes”)**		Yes/No
	Date and hour of event	[Date] and [Hour]
	Degree	Mild Moderate Severe
	Time of onset	Early Late
	Type	ALLERGIC / ALLERGIC-LIKE **Mild** Sparse wheals/itch Skin edema Mild itching / feeling like “velvet in the throat” Nasal congestion Sneezing Conjunctivitis Rhinorrhea **Moderate** Diffuse wheals/intense itch Diffuse skin edema Facial edema without dyspnea Feeling of choking or hoarseness Wheezing/mild bronchospasm without hypoxia **Severe** Dyspnea Erythema—diffuse mucocutaneous symptoms Laryngeal edema with stridor and/or hypoxia Wheezing/bronchospasm Significant hypoxia Anaphylactic shock (severe hypotension and brady-tachyarrhythmia) NON-ALLERGIC **Mild** Mild nausea/limited vomiting Transient chills/heat/redness Headache/dizziness/anxiety/altered taste Slight increase in blood pressure Self-limiting vasovagal reaction **Moderate** Prolonged nausea/vomiting Elevated arterial blood pressure Isolated chest pain Vasovagal reaction **Severe** Treatment-refractory vasovagal reaction Arrythmia Convulsions Severe arterial hypertension CONTRAST MEDIUM EXTRAVASATION
	Type of treatment	Wait and see Drug therapy (specify in “Notes” field) Anesthesiologist’s intervention required
	Event resolution	Spontaneous After treatment After hospitalization Other [free text]
	Notes	[free text]

**Table A6:** REPORT.

FIELD	DETAIL	ADMITTED VALUES	
FEATURES OF PRIMARY TUMOR			
**Lung**		Right Left	
**Lobe**		(if right lung) Upper Middle Lower (if left lung) Upper Lower	
**Site (note: peripheral = outer third of lung; central = inner two-thirds of lung)**		Peripheral Central	
**Segment**		RIGHT LUNG **Upper lobe** 1. Apical 3. Anterior 2. Posterior **Middle lobe** 4. Lateral 5. Medial **Lower lobe** 6. Superior 8. Anterior 9. Lateral 7. Medial 10. Posterior LEFT LUNG **Upper lobe** 1+2.Apicoposterior 3. Anterior 4. Superior lingular 5. Inferior lingular **Lower lobe** 6. Superior 7+8. Anteromedial 9. Lateral 10. Posterior	
**Margins**		Regular Lobulated Irregular Spiculated	
**Density**		Solid Partly solid Ground glass	
**Contrast enhancement**		Nonsignificant (<15-20 HU) Homogeneous Inhomogeneous Necrotic/colliquative components	
**Cavitation** **(visible only if “Yes”)**		Yes/No	
	Maximum diameter	[Numeric] (cm)	
**Bubble-like air space**		Yes/No	
**Air bronchogram/bronchiologram**		Yes/No	
**Maximum diameter**	Measurement plane	Axial Coronal Sagittal	
	Size	≤1 cm >1 cm, ≤2 cm >2 cm, ≤3 cm >3 cm, ≤4 cm >4 cm, ≤5 cm >5 cm, ≤7 cm >7 cm	
	(if maximum diameter >7 cm)	Exact diameter [numeric] (cm)	
**Maximum diameter of solid component (if “Partly solid” density)**	Measurement plane	Axial Coronal Sagittal	
	Maximum diameter	[Numeric] (cm) (if ≤3 cm)	
**Volume**		[Numeric] (cm^3^)	
**LOCOREGIONAL EXTENT**			
**Relationship with neighboring structures** **(visible only if “Yes” and repeatable)**		Yes/No	
	Site	Airways (multiple choice) - Right upper lobe bronchus - Left upper lobe bronchus - Right lower lobe bronchus - Left lower lobe bronchus - Intermediate bronchus - Middle lobe bronchus - Main right bronchus - Main left bronchus - Carina - Trachea Obstructive atelectasis/pneumonia Pulmonary arteries - Right upper lobe artery - Left upper lobe artery - Interlobar artery - Middle lobe artery - Right lower lobe artery - Left lower lobe artery - Right pulmonary artery (extrapericardial tract) - Left pulmonary artery (extrapericardial tract) Pulmonary veins - Right upper lobe vein - Left upper lobe vein - Middle lobe vein - Right lower lobe vein - Left lower lobe vein Pleura - Fissural pleura - Costal pleura - Diaphragmatic pleura Thoracic wall (incl. Pancoast tumor) Mediastinum: - Mediastinal fat - Pericardium - Heart - Aorta/Supraaortic vessels - Right pulmonary artery (intrapericardial tract) - Left pulmonary artery (intrapericardial tract) - Main pulmonary artery - Superior vena cava - Esophagus - Vertebrae (specify if cervical, dorsal; specify which of the following) - body - posterior lamina - vertebral canal/dural sac Diaphragm	
	Type	Airways: - contact - infiltration/occlusion Obstructive atelectasis / pneumonia - sublobar - lobar - whole lung Pulmonary arteries: - contact (<90°) - indeterminate (between >90° and < 360°) - infiltration (360° or intravascular tumor tissue) Pulmonary veins: - contact (<90°) - indeterminate (between >90° and < 360°) - infiltration (360° or intravascular tumor tissue) Pleura: - connection bands - contact (<3 cm) - indeterminate (contact >3 cm) - infiltration (pleural thickening + contact >3 cm and/or obtuse tissue angles) Thoracic wall (incl. Pancoast tumor) - contact (<3 cm) - indeterminate (contact >3 cm) - infiltration of extraparietal soft tissues - osteolysis (specify site) Mediastinum: - Mediastinal fat - Pericardium - Heart - Aorta/supraaortic vessels - Right pulmonary artery (intrapericardial tract) - Left pulmonary artery (intrapericardial tract) - Main pulmonary artery - Superior vena cava - Esophagus contact (<90°) indeterminate (between >90° and <360°) infiltration (360° or intravascular tumor tissue) Infiltration of nervous structures: - Raised right hemidiaphragm - Raised left hemidiaphragm - Extensive involvement of right upper-mid right mediastinum - Extensive involvement of left upper-mid mediastinum - Diaphragm - contact - indeterminate - transdiaphragmatic infiltration	
**Pleural effusion** (v **isible only if “Yes”)**		Yes/No	
	Site	Right Left Bilateral	
	Degree	Mild Moderate Massive	
	Thickness	[Numeric] (cm)	
**Pericardial effusion** **(v** **isible only if “Yes”)**	Site		
	Degree	Mild Moderate Massive	
	Thickness	[Numeric] (cm)	
**LYMPH NODES**			
**Lymph nodes** **(visible only if “Yes” and repeatable)**		Yes/No	
	Type	Nonsignificant (short axis ≤1 cm) Suspicious (short >1 cm) Pathological (necrosis, extracapsular invasion) Confluent lymphoadenopathies (“bulky”)	
	Maximum short axis diameter	[Numeric] (cm)	
	Lymph node stations	supraclavear/low cervical (1) - right (1R) - left (1L) - bilateral (1R and 1L) Upper paratracheal (2) - right (2R) - left (2L) - bilateral (2R and 2L) Lower paratracheal (4) - right(4R) - left (4L) - bilateral (4R and 4L) prevascular (3a) retrotracheal (3p) subaortic (5) paraaortic (6) subcarinal (7) hilar (10) - right (10R) - left (10L) - bilateral (10R and 10L) other [free text]	
	Number of discrete lymph nodes	[Numeric]	
	Number of involved lymph node stations	[Numeric]	
**METASTASES**			
	• Parenchymal nodules	Yes/No Number	
	(if “Yes”, open 2 dialog boxes for each target lesion according to RECIST 1.1 criteria)	Right/left lung Site (lobe and segment) Size (mm) Structure (solid/partly solid/ground glass/cavitated)	
	• Lymphangitic carcinomatosis	Yes/No Right lung/left lung/both lungs Site (lobes)	
	• Pleural metastases	Yes/No Right lung/left lung/both lungs Site (lobe and segment)	
	• Pericardial metastases	Yes/No	
	• Extrathoracic lymph nodes	Yes/No Number of involved lymph node stations	
	(if “Yes”, open 2 dialog boxes for each target lesion according to RECIST 1.1 criteria)	Site [free text] Size (mm) * (* note: if short axis > 15 mm) Structure [free text]	
	• Bone metastases	Yes/No Number of bone segments	
	(if “Yes”, open 2 dialog boxes for each target lesion according to RECIST 1.1 criteria)	Site [free text] Size (mm) * (* note: if size of extraosseous tissue component >10 mm) Structure [free text]	
	• Brain metastases	Yes/No Overall number (brain lobes)	
	(if “Yes”, open 2 dialog boxes for each target lesion according to RECIST 1.1 criteria)	Site (brain lobes) Size (mm) Structure [free text]	
	• Liver metastases	Yes/No Number	
	(if “Yes”, open 2 dialog boxes for each target lesion according to RECIST 1.1 criteria)	Site (liver segments) Size (mm) Structure [free text]	
	• Adrenal metastases	Yes/No Number	
	(if “Yes”, open 2 dialog boxes for each target lesion according to RECIST 1.1 criteria)	Site (right/left adrenal gland) Size (mm) Structure [free text]	
	• Other sites	Yes/No	
	(if “Yes”, open 2 dialog boxes for each target lesion according to RECIST 1.1 criteria)	Site [free text] Size (mm) Structure [free text]	
	Notes	[Free text]	
**Multiple sites of lung involvement (nonmetastatic)**			
**Second lung tumor (synchronous and distinct tumors)** **(v** **isible only if “Yes”)** **(The same features used to classify primary lung tumor apply)**		Yes/No	
	Lung		Right Left
	Lobe		(if right lung) Upper Middle Lower (if left lung) Upper Lower
	Site (note: peripheral = outer third of lung; central = inner two-thirds of lung)		Peripheral Central
	Segment		RIGHT LUNG **Upper lobe** 1. Apical 3. Anterior 2. Posterior **Middle lobe** 4. Lateral 5. Medial **Lower lobe** 6. Superior 8. Anterior 9. Lateral 7. Medial 10. Posterior LEFT LUNG **Upper lobe** 1+2.Apicoposterior 3. Anterior 4. Superior lingular 5. Inferior lingular **Lower lobe** 6. Superior 7 + 8. Anteromedial 9. Lateral 10. Posterior
	Margins		Regular Lobulated Irregular Spiculated
	Density		Solid Partly solid Ground glass
	Contrast enhancement		Nonsignificant (<15–20 HU) Homogeneous Inhomogeneous Necrotic/colliquative components
	Cavitation (visible only if “Yes”)	Yes/No	
		Maximum diameter	[Numeric] (cm)
	Bubble-like air space	Yes/No	
	Air bronchogram/bronchiologram	Yes/No	
	Maximum diameter	Measurement plane	Axial Coronal Sagittal
		Size	≤1 cm >1 cm, ≤2 cm >2 cm, ≤3 cm >3 cm, ≤4 cm >4 cm, ≤5 cm >5 cm, ≤7 cm >7 cm
	(if maximum diameter >7 cm)		Exact diameter [numeric] (cm)
	Maximum diameter of solid component (if “Partly solid” density)	Measurement plane	Axial Coronal Sagittal
		Size	[Numeric] (cm) (if ≤3 cm)
	Volume		[Numeric] (cm^3^)
**Multifocal pattern with multiple ground glass and/or partly solid nodules** **(v** **isible only if “Yes”)**		Yes/No	
	Site	[specify]	
	Measurement plane	Axial Coronal Sagittal	
	Maximum diameter of main (larger) lesion	[Numeric] (cm)	
**Multifocal pattern with pneumonic appearance (i.e., bilateral ground glass and/or patchy consolidation areas) (single tumor, diffuse lung involvement)** **(v** **isible only if “Yes”)** **(The same features used to classify primary lung tumor apply)**		Yes/No	
	Lung	Right (specify lobe(s) and segment(s)) Left (specify lobe(s) and segment(s)) Bilateral (specify lobes and segments)	
	Margins		Regular Lobulated Irregular Spiculated
	Density		Solid Partly solid Ground glass
	Contrast enhancement		Nonsignificant (<15–20 HU) Homogeneous Inhomogeneous Necrotic/colliquative components
	Cavitation (visible only if “Yes”)	Yes/No	
		Maximum diameter	[Numeric] (cm)
	Bubble-like air space	Yes/No	
	Air bronchogram/bronchiologram	Yes/No	
**OTHER FINDINGS**			
**Incidental findings**	Pulmonary	Emphysema (if Yes, type of lesions) Fibrosis (if Yes, type of pattern) Combined forms (fibrosis-emphysema) Other [Free text]	
	Extrapulmonary, thoracic	[Free text]	
	Extrathoracic	[Free text]	
**CONCLUSIONS**			
**Variation with respect to previous imaging examinations**	Yes/No		
	Notes	[Free text]	
**Clinical staging of primary tumor**		cT, N, M (8th Edition, AJCC-UICC 2017)	
		Stage [Alphanumeric]	
**Clinical staging in MULTIPLE SITES OF LUNG INVOLVEMENT**	Second lung tumor (distinct synchronous tumors)	Separate clinical staging for each tumor (8th Edition, AJCC-UICC 2017) (cT, N, M) Stage [Alphanumeric]	
	Multifocal pattern with multiple ground glass and/or partly solid nodules	Clinical staging (8th Edition, AJCC-UICC 2017) (cT main lesion, N single, M single) Stage [Alphanumeric]	
	Multifocal pattern with pneumonic appearance (i.e., bilateral ground glass and/or patchy consolidation areas) (single tumor, diffuse lung involvement)	Clinical staging (8th Edition, AJCC-UICC 20179 (cT based on size, or T3 if in one lobe, or T4 if in more lobes of the same lung, respectively, N single, M single) Stage [Alphanumeric]	
**Further investigations**	Type	PET-CT Biopsy Other [free text]	
**Annotations and comments**		[Free text]	

**Table A7:** IMAGES.

FIELD	DETAIL	ADMITTED VALUES
**Significant key images**		[Images]

## Appendix B

**Table A8:** PATIENT’S CLINICAL DATA (imported from RIS).

FIELD	DETAIL	ADMITTED VALUES
ANTHROPOMETRIC DATA		
**Weight**		[Numeric] (kg)
**Height**		[Numeric] (cm)
**BMI**		[Numeric] (automatically calculated)
**BSA**		[Numeric] (automatically calculated)
**Age**		[Numeric] (years)
**Age range**		0–49 50–69 ≥70
**PERSONAL EVALUATION**		
**Family history of lung cancer** **(visible only if “Yes” and repeatable)**		Yes/No
	Degree of kinship	Mother (1st degree) Father (1st degree)
	Notes	[Free text]
**Family history of cancer** **(visible only if “Yes” and repeatable)**		Yes/No
	Degree of kinship	Mother (1st degree) Father (1st degree)
	Notes	[Free text]
**Predisposing diseases** **(visible only if “Yes” and repeatable)**		Yes/No
	Type	Emphysema COPD Idiopathic pulmonary fibrosis Familial fibrosis Fibrosis in connectivitis Previous cancer (incl. lung cancer, >5 years) Previous chest radiation therapy
	Notes	[Free text]
**Previous lung cancer** **(visible only if “Yes” and repeatable)**		Yes/No
	Histopathological classification (WHO 2015)	Adenocarcinoma Squamous cell carcinoma Large cell carcinoma Neuroendocrine tumors: - Small-cell lung carcinoma; - Large-cell neuroendocrine carcinoma; - typical carcinoid; - atypical carcinoid Adeno-squamous carcinoma Sarcomatoid carcinoma
	pT, N, M, Stage yT, N, M, Stage AJCC-UICC Classification (8th Edition, 2017)	
	Notes	[Free text]
**Risk factors**		Yes/No
	Type	Age (≥ 50 years old) Smoke Secondhand smoke Environmental exposure to the following: - Asbestos; - Radon; - Uranium; - Silica; - Cadmium; - Arsenic; - Beryllium; - Chromium; - Nickel; - Polycyclic aromatic hydrocarbons; - Ethylene oxide; - Benzene; - Coal smoke; - Chloromethylether; - Vinyl chloride; - Fine powders (particulate matter: PM10, PM2.5)
	SMOKING DETAILS	
	Smoker (visible only if “Smoke” selected)	Current smoker Former smoker
	Cigarette smoke	Yes/No
	Number of daily cigarettes (if current smoker)	Light (<15) Heavy (≥15)
	Smoking years	Numeric
	Years from cessation (if former smoker)	≤15 >15
	Cigarettes per year (pack-year) (if former or current smoker)	Numeric (automatically calculated) (N of daily cigarettes x smoking years/20)
	Risk	Low risk (<50 years old and/or <20 pack-years of smoking) Moderate risk (≥50 years old and ≥20 packs per year of active or passive smoking, without other risk factors) High risk **Group 1** (≥50 years old and ≥20 packs per year of smoking history, in presence of another risk factor (except secondhand smoking)) **Group 2** (55–77 years old and ≥30 packs per year of active smoking and smoking cessation <15 years) **Source: NCCN guidelines v. 1.2020**
	Vaping	Yes/No
	Number of daily electronic cigarettes refills (if vaping = Yes)	Numeric
	Number of years (if vaping = Yes)	Numeric
	Notes	[Free text]
**ALLERGY/PREVIOUS OR ONGOING ADVERSE REACTIONS**		
**Allergies** **(visible only if “Yes”)** **(repeatable n times, for all n drugs and/or contrast media specified in “Type”)**		Yes/No
	Type	Drug-related (n of drugs) Contrast medium-related (n of contrast media) Drug-unrelated
	Active principle/molecule (if drug- or contrast medium-related allergy)	[free text]
	Commercial name (if drug- or contrast medium-related allergy)	[free text]
	Notes	[free text]
**PREVIOUS adverse reactions** **(visible only if “Yes” and repeatable)**	Yes/No	
	Date	month/year (mm/yyyy)
	Description	[free text]
	Degree	Mild Moderate Severe
	Time of onset	Early Late
	Notes	[free text]

**Table A9:** CLINICAL EVALUATION.

FIELD	DETAIL	ADMITTED VALUES
CLINICAL INFORMATION		
**Prior examinations** **(visible only if “Yes”)**		Yes/No
	Note	[free text]
**Biological work-up**		Yes/No
	Detail	EGFR mutation KRAS mutation ALK rearrangement (fusion) ROS1 rearrangement (fusion) RET rearrangement (fusion) BRAF V600 mutation PDL-1 expression Immunohistochemistry: TTF-1 CK7 (cytokeratin) Napsin p63, p40 CK5/6 Desmocollin-3 Chromogranin Synaptophysin
**Symptoms**	Type	SPECIFIC SYMPTOMS (for primary tumor) Cough Shortness of breath Chest pain Hemoptysis NONSPECIFIC SYSTEMIC SYMPTOMS Anorexia Weight loss Fatigue Fever SYMPTOMS RELATED TO METASTATIC SITES [Free text]
	Notes	[Free text]
**Clinical indication**		Primary staging Restaging after surgery Restaging after neoadjuvant chemo-radiation therapy Restaging after chemotherapy Restaging after percutaneous ablation Restaging after stereotactic ablation

**Table A10:** IMAGING PROTOCOL.

FIELD	DETAIL	ADMITTED VALUES
IMAGING DATA		
**Date of examination**		[Date]
**Antiallergic premedication**		Yes/No
	Treatment	Steroid Antihistamine
	Complete	Yes/No
	Notes	[free text]
**Nephroprotective protocol**		Yes/No
	Complete	Yes/No
	Serum creatinine	[Numeric] (mg/dL)
	GFR (Glomerular Filtration Rate)	[Numeric] (mL/min) https://www.merckmanuals.com/medical-calculators/GFR_CKD_EPI-it.htm accessed on 20 April 2021 (sex, race, age, serum creatinine level)
	Notes	[free text]
**CONTRAST MEDIUM**		
**Use of contrast medium** **(visible only if “Yes”)**		Yes/No
	Active principle	[Name of active principle of contrast medium]
	Commercial name	[Free text]
	Dose	[Numeric] (mL)
	Flow rate	[Numeric] (mL/s)
	Concentration	[Numeric] (mg I/mL)
	Notes	[Free text]
**ADVERSE EVENTS**		
**ONGOING adverse events** **(visible only if ”Yes”)**		Yes/No
	Date and hour of event	[Date] and [Hour]
	Degree	Mild Moderate Severe
	Time of onset	Early Late
	Type	ALLERGIC / ALLERGIC-LIKE **Mild** Sparse wheals/itch Skin edema Mild itching/feeling like “velvet in the throat” Nasal congestion Sneezing Conjunctivitis Rhinorrhea **Moderate** Diffuse wheals/intense itch Diffuse skin edema Facial edema without dyspnea Feeling of choking or hoarseness Wheezing / mild bronchospasm without hypoxia **Severe** Dyspnea Erythema—diffuse mucocutaneous symptoms Laryngeal edema with stridor and/or hypoxia Wheezing / bronchospasm Significant hypoxia Anaphylactic shock (severe hypotension and brady-tachyarrhythmia) NON-ALLERGIC **Mild** Mild nausea/limited vomiting Transient chills/heat/redness Headache/dizziness/anxiety/altered taste Slight increase in blood pressure Self-limiting vasovagal reaction **Moderate** Prolonged nausea/vomiting Elevated arterial blood pressure Isolated chest pain Vasovagal reaction **Severe** Treatment-refractory vasovagal reaction Arrythmia Convulsions Severe arterial hypertension
	Type of treatment	Wait and see Drug therapy (specify in “Notes” field) Anesthesiologist’s intervention required
	Event resolution	Spontaneous After treatment After hospitalization Other [free text]
	Notes	[free text]

**Table A11:** REPORT.

FIELD	DETAIL	ADMITTED VALUES	
FEATURES OF PRIMARY TUMOR			
**Lung**		Right Left	
**Lobe**		(if right lung) Upper Middle Lower (if left lung) Upper Lower	
**Site**		Peripheral Central	
**Segment**		RIGHT LUNG **Upper lobe** 1. Apical 3. Anterior 2. Posterior **Middle lobe** 4. Lateral 5. Medial **Lower lobe** 6. Superior 8. Anterior 9. Lateral 7. Medial 10. Posterior LEFT LUNG **Upper lobe** 1+2.Apicoposterior 3. Anterior 4. Superior lingular 5. Inferior lingular **Lower lobe** 6. Superior 7 + 8. Anteromedial 9. Lateral 10. Posterior	
**Margins**		Regular Lobulated Irregular Spiculated	
**Density**		Solid Partly solid Ground glass	
**Contrast enhancement**		Nonsignificant (<15-20 HU) Homogeneous Inhomogeneous Necrotic/colliquative components	
**Cavitation** **(visible only if “Yes”)**		Yes/No	
	Maximum diameter	[Numeric] (cm)	
**Bubble-like air space**		Yes/No	
**Air bronchogram/bronchiologram**		Yes/No	
**Maximum diameter**	Measurement plane	Axial Coronal Sagittal	
	Size	≤1 cm >1 cm, ≤2 cm >2 cm, ≤3 cm >3 cm, ≤4 cm >4 cm, ≤5 cm >5 cm, ≤7 cm >7 cm	
**Maximum diameter of solid component (if “Partly solid” density)**	Measurement plane	Axial Coronal Sagittal	
	Maximum diameter	[Numeric] (cm) (if ≤3 cm)	
**Volume**		[Numeric] (cm^3^)	
**LOCOREGIONAL EXTENT**			
**Relationship with neighboring structures** **(visible only if “Yes” and repeatable)**		Yes/No	
	Site	Airways (multiple choice) - Right upper lobe bronchus - Left upper lobe bronchus - Right lower lobe bronchus - Left lower lobe bronchus - Intermediate bronchus - iddle lobe bronchus - Main right bronchus - Main left bronchus - Carina - Trachea Obstructive atelectasis/pneumonia Pulmonary arteries - Right upper lobe artery - Left upper lobe artery - Interlobar artery - Middle lobe artery - Right lower lobe artery - Left lower lobe artery - Right pulmonary artery (extrapericardial tract) - Left pulmonary artery (extrapericardial tract) Pulmonary veins - Right upper lobe vein - Left upper lobe vein - Middle lobe vein - Right lower lobe vein - Left lower lobe vein Pleura - Fissural pleura - Costal pleura - Diaphragmatic pleura Thoracic wall (incl. Pancoast tumor) Mediastinum - Mediastinal fat - Pericardium - Heart - Aorta/Supraaortic vessels - Right pulmonary artery (intrapericardial tract) - Left pulmonary artery (intrapericardial tract) - Main pulmonary artery - Superior vena cava - Esophagus - Nervous structures: - Right phrenic nerve - Left phrenic nerve - Right recurrent laryngeal nerve - Left recurrent laryngeal nerve - Vertebrae (specify if cervical, dorsal; specify which of the following) - body - posterior lamina - vertebral canal/dural sac Diaphragm	
	Type	Airways: - contact - infiltration/occlusion Obstructive atelectasis / pneumonia - sublobar - lobar - whole lung Pulmonary arteries: - contact (<90°) - indeterminate (between >90° and <360°) - infiltration (360° or intravascular tumor tissue) Pulmonary veins: - contact (<90°) - indeterminate (between >90° and <360°) - infiltration (360° or intravascular tumor tissue) Pleura: - connection bands - contact (<3 cm) - indeterminate (contact > 3 cm) - infiltration (pleural thickening + contact >3 cm and/or obtuse tissue angles) Thoracic wall (incl. Pancoast tumor) - contact (<3 cm) - indeterminate (contact > 3 cm) - infiltration of extraparietal soft tissues - osteolysis (specify site) Mediastinum: - Mediastinal fat - Pericardium - Heart - Aorta/Supraaortic vessels - Right pulmonary artery (intrapericardial tract) - Left pulmonary artery (intrapericardial tract) - Main pulmonary artery - Superior vena cava - Esophagus contact (<90°) indeterminate (between >90° and <360°) infiltration (360° or intravascular tumor tissue) Infiltration of nervous structures: - Raised right hemidiaphragm - Raised left hemidiaphragm - Extensive involvement of right upper-mid right mediastinum - Extensive involvement of left upper-mid mediastinum Diaphragm - contact - indeterminate - transdiaphragmatic infiltration	
**Pleural effusion** **(v** **isible only if “Yes”)**		Yes/No	
	Site	Right Left Bilateral	
	Degree	Mild Moderate Massive	
	Thickness	[Numeric] (cm)	
**Pericardial effusion** **(v** **isible only if “Yes”)**	Site		
	Degree	Mild Moderate Massive	
	Thickness	[Numeric] (cm)	
**LYMPH NODES**			
**Lymph nodes** **(visible only if “Yes” and repeatable)**		Yes/No	
	Type	Nonsignificant (short axis ≤ 1 cm) Suspicious (short >1 cm) Pathological (necrosis, extracapsular invasion) Confluent lymphoadenopathies (“bulky”)	
	Maximum short axis diameter	[Numeric] (cm)	
	Lymph node stations	supraclavear/low cervical (1) - right (1R) - left (1L) - bilateral (1R and 1L) Upper paratracheal (2) - right (2R) - left (2L) - bilateral (2R and 2L) Lower paratracheal (4) - right(4R) - left (4L) - bilateral (4R and 4L) prevascular (3a) retrotracheal (3p) subaortic (5) paraaortic (6) subcarinal (7) hilar (10) - right (10R) - left (10L) - bilateral (10R and 10L) other [free text]	
	Number of discrete lymph nodes	[Numeric]	
	Number of involved lymph node stations	[Numeric]	
**METASTASES**			
	Type	Pulmonary metastatic nodules Lymphangitic carcinomatosis Pleural metastases Pericardial metastases Extracompartmental lymph nodes Intrathoracic bone metastases (i.e., distant from primary tumor)	
	Measurement plane	Axial Coronal Sagittal	
	Maximum diameter	[Numeric] (cm)	
	Number	[Numeric] or multiple (if more than 5)	
	Site	Pulmonary nodules Same lung lobe as tumor Other lung lobe (specify) Contralateral lung Lymphangitic carcinomatosis Same lung lobe as tumor Other lung lobe (specify) Contralateral lung Pleural metastases Mediastinal pleura Costal pleura Other (specify) Pericardial metastases(specify)	
	Metastases-related effusion	Yes/No	
		Pleural effusion Right Left Bilateral Pericardial effusion Thickness [numeric] (cm)	
**MULTIPLE SITES OF LUNG INVOLVEMENT (nonmetastatic)**			
**Second lung tumor (synchronous and distinct tumors)** **(v** **isible only if “Yes”)** **(The same features used to classify primary lung tumor apply)**		Yes/No	
	Lung		Right Left
	Lobe		(if Right lung) Upper Middle Lower (if Left lung) Upper Lower
	Site		Peripheral Central
	Segment		RIGHT LUNG **Upper lobe** 1. Apical 3. Anterior 2. Posterior **Middle lobe** 4. Lateral 5. Medial **Lower lobe** 6. Superior 8. Anterior 9. Lateral 7. Medial 10. Posterior LEFT LUNG **Upper lobe** 1 + 2. Apicoposterior 3. Anterior 4. Superior lingular 5. Inferior lingular **Lower lobe** 6. Superior 7 + 8. Anteromedial 9. Lateral 10. Posterior
	Margins		Regular Lobulated Irregular Spiculated
	Density		Solid Partly solid Ground glass
	Contrast enhancement		Nonsignificant (<15–20 HU) Homogeneous Inhomogeneous Necrotic/colliquative components
	Cavitation (visible only if “Yes”)	Yes/No	
		Maximum diameter	[Numeric] (cm)
	Bubble-like air space	Yes/No	
	Air bronchogram/bronchiologram	Yes/No	
	Maximum diameter	Measurement plane	Axial Coronal Sagittal
		Size	≤1 cm >1 cm, ≤2 cm >2 cm, ≤3 cm >3 cm, ≤4 cm >4 cm, ≤5 cm >5 cm, ≤7 cm >7 cm
	Maximum diameter of solid component (if “Partly solid” density)	Measurement plane	Axial Coronal Sagittal
		Size	[Numeric] (cm) (if ≤3 cm)
	Volume		[Numeric] (cm^3^)
**Multifocal pattern with multiple ground glass and/or partly solid nodules** **(v** **isible only if “Yes”)**		Yes/No	
	Site	(specify)	
	Measurement plane	Axial Coronal Sagittal	
	Maximum diameter of main (larger) lesion	[Numeric] (cm)	
**Multifocal pattern with pneumonic appearance (i.e., bilateral ground glass and/or patchy consolidation areas) (single tumor, diffuse lung involvement)** **(v** **isible only if “Yes”)** **(The same features used to classify primary lung tumor apply)**		Yes/No	
	Lung	Right Left	
	Lobe		(if Right lung) Upper Middle Lower (if Left lung) Upper Lower
	Site		Peripheral Central
	Segment		RIGHT LUNG **Upper lobe** 1. Apical 3. Anterior 2. Posterior **Middle lobe** 4. Lateral 5. Medial **Lower lobe** 6. Superior 8. Anterior 9. Lateral 7. Medial 10. Posterior LEFT LUNG **Upper lobe** 1+2.Apicoposterior 3. Anterior 4. Superior lingular 5. Inferior lingular **Lower lobe** 6. Superior 7+8. Anteromedial 9. Lateral 10. Posterior
	Margins		Regular Lobulated Irregular Spiculated
	Density		Solid Partly solid Ground glass
	Contrast enhancement		Nonsignificant (<15-20 HU) Homogeneous Inhomogeneous Necrotic/colliquative components
	Cavitation (visible only if “Yes”)	Yes/No	
		Maximum diameter	[Numeric] (cm)
	Bubble-like air space	Yes/No	
	Air bronchogram/bronchiologram	Yes/No	
	Maximum diameter	Measurement plane	Axial Coronal Sagittal
		Size	≤1 cm >1 cm, ≤2 cm >2 cm, ≤3 cm >3 cm, ≤4 cm >4 cm, ≤5 cm >5 cm, ≤7 cm >7 cm
	Maximum diameter of solid component (if “Partly solid” density)	Measurement plane	Axial Coronal Sagittal
		Size	[Numeric] (cm) (if ≤3 cm)
	Volume		[Numeric] (cm^3^)
**OTHER FINDINGS**			
**Incidental findings**	Pulmonary	[Free text]	
	Extrapulmonary, thoracic	[Free text]	
	Extrathoracic	[Free text]	
**CONCLUSIONS**			
**Variation with respect to previous imaging examinations**	Yes/No		
	Notes	[Free text]	
**Clinical staging of primary tumor**		cT, N, M (8th Edition, AJCC-UICC 2017)	
		Stage [Alphanumeric]	
**Clinical staging in MULTIPLE SITES OF LUNG INVOLVEMENT**	Second lung tumor (distinct synchronous tumors)	Separate clinical staging for each tumor (8th Edition, AJCC-UICC 2017) (cT, N, M) Stage [Alphanumeric]	
	Multifocal pattern with multiple ground glass and/or partly solid nodules	Clinical staging (8th Edition, AJCC-UICC 2017) (cT main lesion, N single, M single) Stage [Alphanumeric]	
	Multifocal pattern with pneumonic appearance (i.e., bilateral ground glass and/or patchy consolidation areas) (single tumor, diffuse lung involvement)	Clinical staging (8th Edition, AJCC-UICC 2017) (cT based on size, or T3 if in one lobe, or T4 if in more lobes of the same lung, respectively, N single, M single) Stage [Alphanumeric]	
**Further investigations**	Type	PET-CT Bronchoscopy (EBUS-FNA/Biopsy) Endoscopy (EUS-FNA/Biopsy) Trans-thoracic biopsy (TTB) Other [free text]	
**Annotations and comments**		[Free text]	

**Table A12:** IMAGES.

FIELD	DETAIL	ADMITTED VALUES
**Significant key images**		[Images]
